# Introducing quality clusters in general practice – a qualitative study of the experiences of cluster coordinators

**DOI:** 10.1186/s12875-022-01828-2

**Published:** 2022-08-25

**Authors:** Marius Brostrøm Kousgaard, Thorbjørn Hougaard Mikkelsen, Maria Bundgaard, Marie Henriette Madsen, Morten Bonde Klausen, Mads Toft Kristensen, Pia Kürstein Kjellberg, Jens Søndergaard

**Affiliations:** 1 The Research Unit for General Practice and Section of General Practice, Department of Public Health, University of Copenhagen, Øster Farimagsgade 5, 1014 Copenhagen K, Denmark; 2 Research Unit of General Practice, Institute of Public Health, University of Southern Denmark, J.B. Winsløws Vej 9A, 1, 5000 Odense C, Denmark; 3 Research Unit of Emergency Medicine, Department of Regional Health Research, University of Southern Denmark, Odense, Denmark; 4 Emergency Department, Hospital Sønderjylland, Kresten Philipsens vej 15, indgang F, 6200 Aabenraa, Denmark; 5 Research Unit of General Practice, Department of Public Health, University of Southern Denmark, J.B. Winsløws Vej 9A, 1, 5000 Odense C, Denmark; 6 VIVE The Danish Center for Social Science Research, Herluf Trolles Gade 11, 1052 Copenhagen K, Denmark

**Keywords:** Clusters, Challenges, Enablers, General practice, Quality improvement, Qualitative study

## Abstract

**Background:**

In 2018, the concept of clusters was introduced as a new model for data-driven quality improvement in general practice in Denmark. However, there is little research on the development and implementation of general practice clusters. The study explores how the cluster coordinators responsible for leading the clusters forward enacted and experienced their role during the early years of the clusters with attention to the challenges and enablers perceived in the process.

**Methods:**

Qualitative, semi-structured interviews with 25 cluster coordinators from clusters that had carried out at least two meetings on a specific professional topic. The coordinators represented clusters of varying sizes and different geographic locations. Key topics in the interview guide were the development and structure of the cluster, the role of the coordinator, obtainment of data for the meetings, the role of external support, the form and content of the meetings, the participation and engagement of the members. A thematic analysis – shaped by the original aims and categories of the study while also being open to emerging themes – was performed on the transcribed interview material.

**Results:**

Important enablers in the process of developing the clusters included the positive engagement of the GPs, the support offered by regional quality units and a national quality organisation for general practice, and the funding provided by the formal cluster framework. Challenges initially included setting up the clusters administratively and translating the open cluster concept into a local, workable model; and later obtaining relevant data for the cluster meetings and facilitating peer discussions about the data.

**Conclusion:**

The coordinators generally experienced that the development of the clusters had progressed relatively fast with engagement from most of the participating GPs. Still, challenges with data obtainment, data analysis, and facilitation will have to be addressed ongoingly. Future research should investigate learning processes at the cluster meetings and how the clusters impact clinical practice and collaborative relations between general practice and other health care providers.

**Supplementary Information:**

The online version contains supplementary material available at 10.1186/s12875-022-01828-2.

## Background

In 2018, the concept of clusters was introduced as a new model for quality improvement for general practice in Denmark. The basic idea was that the general practitioners (GPs) should form relatively large groups (clusters) based on geographical proximity, where they should meet regularly (two to four times a year) and work with data-driven quality improvement. The cluster model followed a previous national model for quality improvement, which was based on mandatory accreditation, and the differences between the two approaches were noticeable. In accreditation, the areas to be addressed were predefined by the accreditation standards, the process was centrally governed by a national organisation for accreditation, and the general practice clinics were assessed by representatives from this external organisation [1, 2]. In contrast, the GP clusters should be self-organizing and each cluster would have the freedom to choose its own focus areas, and although the collective agreement between the Organisation of General Practitioners and the Danish Regions applied some regulative pressure to induce participation in the clusters (see below), the collective agreement emphasized that the work of the clusters should be separated from formal mechanisms of control in order to ensure the best conditions for a learning-oriented culture. A move towards less standardized and more decentralized approaches to quality improvement in general practice has also been seen in the primary care systems of Scotland and Wales [3–5]. The Scottish model was inspired by experiences from so called ‘quality circles’ in Europe and elsewhere; i.e., small, local groups where GPs get together regularly for reflections and discussions about potential improvements of their practice [6, 7]. Research has shown that GPs are positive about quality circles where they may engage in collective processes of learning in relation to professional development and quality improvement and support each other in their professional life [7]. While the cluster model in Denmark has some similarities with quality circles, i.e. a focus on learning and quality improvement, autonomy to decide which issues to address, use of feedback and peer-discussions [7], there are also important differences. First, there is a difference in size; whereas quality circles “comprise small groups of 6–12 health care professionals” [7], most clusters in Denmark are much larger having an average size of 29 GPs (cf. the section below on the framework of the quality clusters). Second, the clusters are part of a national strategy and a formal framework for quality improvement in general practice that includes technical and inspirational support from a central quality or0067anisation (Quality in General Practice – KiAP), and five regional quality units as well as increased funding and attention.

So far, little research has been published about the development, implementation, internal processes and impact of GP-clusters. In Wales, where the clusters were introduced in 2014, an evaluation found that although the clusters had led to some local improvements, the process had been characterized by administrative implementation challenges and the use of patient data had not been developed sufficiently [4, 5]. In Scotland, a recent survey showed that the GPs had positive experiences with the atmosphere, organization and facilitation at the cluster meetings [8]. However, the majority of the GPs did not report improvements in quality, and the quality leads found that support for activities in the clusters was suboptimal [8].

In the Danish cluster model, so called ‘cluster coordinators’ (similar to the ‘cluster quality leads’ in the Scottish clusters) were given a central role in organising the local clusters. Hence, in the formation process, each cluster was to appoint a cluster coordinator among the GPs in the cluster. According to the collective agreement, the role of the cluster coordinator was to ensure progress in the operation of the clusters, for example by arranging cluster meetings on specific topics, by collecting data for the professional discussions, and by acting as a contact person in relation to external parties. Therefore, this study explored how the cluster coordinators enacted and experienced their role during the early years of the clusters with particular attention to the challenges and enablers perceived in the process.

### Setting

In the tax-financed health care system in Denmark, general practice serves as the first point of contact and as a gatekeeper to secondary medical services. The general practice sector is composed of mostly privately operated clinics; both solo-clinics and partnership clinics (with two or more GPs). GPs are private entrepreneurs who contract with the five administrative regions that are politically responsible for health services in the hospitals and in the practice sector (which is comprised of private health providers such as GPs, practicing specialists, physiotherapists, and psychologists). The association of the administrative regions in Denmark (Danish Regions) negotiates a collective agreement with the Organization of General Practitioners setting the terms of remuneration (a combination of fee-for service and capitation fees), the services provided, and issues of quality assurance and quality improvement. During the last decades, Danish general practice has been involved in various initiatives for quality improvement including clinical guidelines, indicator-based feedback, patient surveys, and, from 2016–2018, accreditation [2, 9, 10]. Apart from the individual GP clinics, these initiatives included a number of institutional actors such as the Danish College of General Practitioners, the Organisation of General Practitioners (PLO), the Danish Regions, the Danish Institute for Quality and Accreditation in Healthcare (IKAS), the Danish Quality Unit of General Practice (DAK-E) (a predecessor to KiAP, see below), and the five regional quality units established in agreement between each of the five administrative regions and the regional PLO-organisations.

### The framework of the quality clusters

The concept of quality clusters was introduced in the collective agreement for general practice 2018–2021. The GPs should form and operate local clusters themselves, and according to the collective agreement, the number of clinics in a cluster should be large enough to encompass at least 30.000 patients, corresponding to 16–20 GPs on average. At the time of this study, the average cluster included 29 GPs and 48.000 patients with the smallest cluster consisting of 10 GPs and the largest of 73 GPs. The GPs were expected to meet regularly (two to four times a year) and work with self-selected topics based on various descriptive data [11]. According to the collective agreement, the clusters could work with health data in a broad sense and various kinds of data were mentioned, e.g. data on chronic care management, medication, patient satisfaction, hospital admissions and readmissions, referrals, and use of municipal services.

Upon establishment each cluster received 10.000 DKK (1.345 EUR) pr. GP and 4,26 DKK (0,57 EUR) yearly pr. patient attached to the clinics in the clusters. This was further supplemented by means from the Foundation of General Practice. Guidance on the use of the financial means of the clusters states that the means can be used to remunerate cluster coordinators, board members, or other individuals with specific tasks in the cluster (with terms being settled in the cluster), and to cover expenses for meeting facilities, catering services, and fees for external speakers or meeting facilitators. While participation in specific quality improvement activities in general practice is sometimes remunerated via the collective agreement, ordinary cluster members (i.e. the GPs) are not remunerated for their participation in cluster meetings or for implementing ideas from the cluster meetings in the clinic.

To support overall quality improvement in general practice, including the cluster model, a new national organization, Quality in General Practice (KiAP) was established and funded in agreement by the Danish Regions and the Organisation of General Practitioners. In relation to the clusters, the role of KiAP was to promote and support the development and operation of the clusters. Hence, KiAP has given administrative advice to the clusters and developed topic specific ‘cluster packages’, i.e. documents on a specific topic with relevance for general practice which includes descriptions of professional guidelines and what data to extract and discuss in the cluster. Examples of topics include conjunctivitis, prescriptions of non-steroidal anti-inflammatory drugs (NSAIDs), diabetes, and chronic obstructive pulmonary disease (COPD). Furthermore, the regional quality units were to offer various forms of support for the clusters, such as cluster packages, data extraction, data interpretation (e.g. in relation to prescription data), and facilitation of meetings. While there is no mandatory training program for the coordinators, some courses and network sessions have been made available to the coordinators by the supporting organisations.

To induce participation in the clusters, the collective agreement stated that clinics, which did not enrol in a cluster, had to take part in a process of accreditation. If the number of clinics that did not enrol in a cluster was too small for running an accreditation program, the situation would be reconsidered. As it turned out, the GPs established and joined the clusters faster than expected, and by November 2019, around 98% of the GPs had joined a cluster [11]. Therefore, it was later decided that the few clinics that had not joined a cluster would receive a visit from a quality consultant and make arrangements about a process of quality assurance. At the beginning of the study, there were approximately 100 clusters in Denmark and at the time of writing there were 114 clusters.

## Methods

The results reported in this paper are based on qualitative interviews with 25 cluster coordinators (i.e. active GPs who have been elected as coordinators by their colleagues in the cluster). These interviews were part of a larger qualitative study, which also included background interviews with representatives from the stakeholders (to improve our knowledge of the content and context of the cluster concept) as well as interviews with ordinary cluster members, and observations of cluster meetings. Findings from the interviews with the ordinary cluster members will be reported separately.

### Study participants

In order to ensure that the coordinators had experiences with professional meetings in the cluster, we included participants from clusters that had carried out at least two meetings on a specific topic at the time of the study. Also, we planned to obtain variation in our sample particularly with regards to the characteristics of the clusters represented by the coordinators (geographical location and the size of the clusters). When selecting clusters and coordinators for the study, we used an overview provided by KiAP containing information on all registered clusters in May 2019. At this point in time, 109 clusters had already been officially registered. At the deadline of formation (November 1 2019) 114 clusters had registered. Using this overview, we began to invite coordinators from clusters of varying geographic location and size. Invitations and appointments were handled via e-mail (in some cases supplemented by a telephone call). In case of refusals or exclusion, we contacted new potential participants. When we reached our goal of acceptance from 25 cluster coordinators (five from each of the five regions), we had contacted 30 coordinators. Of the five coordinators we contacted but did not include in the study, the coordinators from two clusters had not yet carried out at least two meetings, two coordinators replied that they did not have time to participate, and one coordinator declined participation without providing a reason. The final sample included five clusters from each of the five regions, and clusters from city areas as well as countryside clusters. The largest cluster in the study included over 50 GPs while the smallest included 10–15 GPs. All clusters represented had carried out between two and six topic-meetings at the time of the study. All participants gave their informed consent to participate in the study.

### Interviews

From October 2019 to February 2020, we carried out qualitative semi-structured interviews with 25 cluster coordinators. Most interviews were performed in the clinic of the coordinator, but some were performed at the venues of the cluster meetings. The interviews lasted 45–90 min and were audio recorded and transcribed. The interview guide was shaped through discussions in the research group and its content was inspired by the central elements in the cluster model (self-organization, the role of the cluster coordinator, using data for improvement), the background interviews with stakeholders, literature on quality circles [6, 7], feedback from the directors of the four Research Units for General Practice in Denmark, and a pilot interview with a cluster coordinator. In relation to this paper, the relevant topics of the interview guide were:

- ◦ The development and structure of the cluster (e.g. process of development and the role of existing professional forums, election of coordinator, board composition, work division, rules of membership)
- ◦ The role of the coordinator (motivation for taking on the role, responsibilities and tasks, challenges and enablers, needs of external support)
- ◦ Selection of topics for the meetings
- ◦ Obtainment of data for the meetings and role of external support
- ◦ The form and content of the professional meetings (typical progress of meetings, topics covered, structuring of the meetings, use of data at the meetings, role of external actors at the meetings, experiences of the meetings)
- ◦ The participation and engagement of the members

### Analysis

First, we read all of the transcribed interviews to obtain overview and increased familiarity [12]. Then we discussed our initial impressions and potential themes for further exploration. Subsequently, we coded the transcribed interviews with reference to the central themes of the interview guide while also being open to emerging themes. In this way, the analysis was both grounded in the empirical data and shaped by the original aims and categories of the research project [12]. Using the coded data material, we then created condensed descriptions of themes and subthemes and we used these as a basis for further discussions about how to interpret and present the data. Quotations from different cluster coordinators are used illustratively. Data was managed in NVivo and Microsoft Word.

Five members of the research team had a background in social science (MBK, THM, MBOK, MHM, PK) while three members were medical doctors (MB, MTK, JS), two of whom were GPs (MTK, JS).

## Results

Overall, the cluster coordinators had been engaged in activities relating to three main areas when developing and operating the clusters: 1) Translation of the general cluster concept into a local cluster model; 2) Administration; 3) Planning, preparing and facilitating the professional meetings in the cluster.

Below, we outline the central activities of the coordinators and identify the challenges and enablers related to these. Further, since the cluster concept relies heavily on the willingness of the GPs to attend and engage in the meetings of the cluster, we also explore how the coordinators experienced and approached issues of attendance and engagement.

### Translating the overall cluster concept into a local model

As mentioned in the introduction, the overall cluster framework was relatively open and did not include detailed descriptions of what the structures and processes of the clusters should look like. Therefore, one of the primary responsibilities of the coordinators was to take the lead in translating the overall cluster concept into a functioning, local cluster. Although the openness of the framework was generally seen as an advantage of the cluster concept due to the autonomy it entailed (cf. below), it also made the translation work more comprehensive for the coordinators. It was the novelty and scale of the task rather than one particular issue, which made this translation work challenging. As one cluster coordinator said:

*It’s a challenge because we have to start from scratch […] invent something... […] we get together in the group and say ‘now we have to invent how to work in clusters – how should that look like?’* (Cluster coordinator)

During the establishment phase, the cluster coordinators were invited to network meetings arranged by KiAP (where ideas and experiences could be shared), and while the coordinators described these meetings as inspirational, they also underlined that the basic structure of their cluster had to be decided upon locally. Some of the issues to be addressed and agreed upon were:

- ◦ The basic organization of the cluster: How to compose the board, whether to employ a secretary, how to allocate tasks between the coordinator(s), the board, and the members.
- ◦ Finances: How to use the funds of the cluster (e.g. in regards to remuneration of the coordinator, the board, secretary assistance, and external expert speakers).
- ◦ Alignment of expectations and ambitions in the cluster in regards to the time investments and workload to be expected of the members (particularly in relation to the frequency and duration of cluster meetings and the preparation work prior to the meetings).
- ◦ How to respond if members did not attend the cluster meetings regularly.
- ◦ The form and content of the cluster meetings.

It was important for the coordinators that the cluster members were included in the discussions about these issues. The coordinators viewed it as a critical part of their job to summarize the preferences of the members and turn these preferences into in a model that would have widespread support in the cluster. Some of the issues above were relatively easily resolved (such as composing the board and selecting topics for the first meetings) while others required more discussions (such as the issue of non-attendance to which we return later).

### Administrative work

One of the first tasks of the coordinators was to set up the cluster administratively. This included official registration of the cluster with the authorities (including the tax authorities), getting a bank account, and finding software solutions to share documents among members of the cluster (such as articles of the association, minutes of meetings, and address lists). Many of the coordinators described this administrative work as troublesome, time demanding and frustrating. Some material guiding the formal establishment of the clusters became available from a temporary central unit, but since the establishment of the clusters took off earlier than expected, the central unit had to develop the material as needs were emerging. For the coordinators this implied that they had to deal with administrative tasks that were not part of their normal professional role and which did not interest them:

*It was very troublesome with many emails back and forth and the tax authorities, and security codes, and a bank account […] I was surprised that I used so ridiculously many hours on this while thinking: why isn’t someone else doing this? It is a waste of my time.* (Cluster coordinator)

However, after the clusters had been formally established, the amount of administrative work had decreased and was experienced as less problematic. The ensuing administrative work consisted of accounting tasks (payment of invoices, annual accounts), planning the general assembly, sending out invitations for the regular cluster meetings, and responding to inquiries from external actors. Some cluster coordinators had delegated some of the administrative tasks to another board member or to a secretary (e.g. from their own clinic) in order to concentrate on other issues in the cluster. Such delegation was enabled by the financial conditions of the clusters.

### Preparation and facilitation of cluster meetings

The coordinators considered the planning, preparation, and facilitation of meetings to be essential activities for ensuring that the cluster meetings became inspirational and useful for the participants.

Planning and preparing for the meetings involved selecting topics for the meetings, contacting potential speakers, making arrangements about venue and catering services, obtaining and making data ready for presentation. Several coordinators mentioned the funding of the clusters as an enabling factor when preparing the meetings since this allowed for selecting suitable venues, for inviting interesting guest speakers (or in some cases professional facilitators), and again for delegating some of the practical work to a secretary. The selection of topics for the meetings usually began with a brainstorm where the members came up with various ideas and suggestions. Subsequently, the coordinator (and the board) selected a topic for the next meeting and explored opportunities for obtaining data and potential external speakers to contact. Several coordinators mentioned that they initially wanted the members to get to know each other and build up trust. Therefore, for the first meetings they preferred topics on which consensus about best professional practices was assumed (e.g. conjunctivitis) rather than topics where disagreements could be expected (e.g. addictive prescription drugs).

Many coordinators described the available cluster packages (designed by KiAP and the regional quality units) as an important source of inspiration when selecting topics. However, in some clusters, the use of cluster packages had been limited since members believed that the selection of topics and data should not be influenced by the governance agenda of external regional or national actors.

For the coordinators, the primary challenges in relation to the meetings concerned the obtainment of data and the facilitation of the discussions at the meetings.

#### Obtainment of data

Many clusters had used quantitative data as a basis for deliberations among the GPs (e.g. prescription data or data on medical services performed by the clinics). Data could be obtained from several sources such as national and regional registers, the participating clinics, or the municipalities. Obtaining data for the meetings was usually the responsibility of the coordinators (or another board member), but for topics within the expertise of the regional quality units such as medication and use of the Shared Medication Record (FMK—the national database on medication), the regional quality consultants could perform the actual extraction of data. This support was considered important by the coordinators and even more so because delays in the updating of the national web portal containing information on medication in general practice (Ordiprax/Ordirax +) had made the collection of prescription data more difficult than planned.

Many clusters also had experiences with letting the members handle the data collection themselves (e.g. by extracting data on specific services or referrals to specialist services from the information system of the clinic). While using data collected by the clinics opened up for a wider range of topics, several coordinators pointed to challenges with this approach. First, the reported data from the clinics were sometimes flawed because not all GPs had sufficient technical knowledge of the specific module for data extraction and/or because the GPs in a cluster were not always able to deliver the same kind of data due to differences in their electronic medical record systems. Second, it could be difficult to secure that all participants remembered to generate and send in the requested data before the meeting:

*I was to present data from 12 of the clinics but only four of them had reported their data [before the meeting]. The rest brought their data to the meeting but that made it very difficult to incorporate these data [into the presentation].* (Cluster coordinator)

Also, in some clusters, the members had made it clear that they did not wish to spend time on collecting data prior to the meetings. For these reasons, some coordinators had decided not to rely on data collected by the clinics.

Apart from focusing on traditional medical topics (such as treatment chronic of diseases), many coordinators expressed an interest in using the clusters to improve collaboration with the municipalities, and some of the clusters had selected topics in this area (e.g. alcohol abuse, medical reports requested by the municipality, referrals to municipal health care services). But while the regional quality units had existed for many years to support quality improvement in general practice, there were no such units in the municipal area (since it is not the municipalities but the regions that are politically responsible for general practice, cf. above). Hence, challenges with obtaining data seemed more prominent for topics related to the municipalities. Here, the coordinators lacked an overview of what kinds of data were available and how to obtain them. The coordinators often found it difficult to get in contact with the right employees in the municipality and make agreements about obtaining data. However, a few coordinators reported that the municipality had proactively engaged in a dialogue about the preparation of data for the cluster meetings.

#### Facilitation of meetings

In terms of conducting the cluster meetings, the data indicated that the role of the coordinators varied. Several coordinators told that they acted as meeting facilitators themselves. Since it could be difficult to facilitate the meetings while also taking part in the professional discussions they were sometimes assisted by a colleague. Other coordinators had delegated facilitation to a board member or an external speaker (e.g. a medicine consultant from the regional quality unit), and in a few clusters, the members took turns in the role of the meeting manager/facilitator.

Many of the coordinators did not seem to be familiar with the use of quality improvement techniques such as the PDSA-Cycle (Plan-Do-Study-Act). However, some coordinators had experiences with meeting management and facilitation from other professional forums (e.g. continuing medical education groups) or teaching. Other coordinators did not have such experiences, and for them it could be a challenge to facilitate the meetings although participation in the network sessions for coordinators arranged by KiAP had provided them with some inspiration.

The coordinators were not expected to act as professional experts in relation to their colleagues. Rather, they worked to ensure that the meetings were planned and executed so that the participants were activated (particularly by using discussions in small groups), that the discussions stayed focused on the topic selected, and that there was an atmosphere of trust between the participants which the coordinators considered to be important for creating a good learning environment. Hence, many coordinators described that they actively encouraged an open and trust-based dialogue at the meetings, for example by exposing their own shortcomings in relation to the given topic:

*I have tried to lead by example. I tell them about my own bad habits […] and when you do that you get a different response. Then somebody else says: ‘God, I do that too!’ and then it’s not so embarrassing. So being able to expose yourself and talk about your bad habits, that creates a sense of trust...* (Cluster coordinator)

Generally, the coordinators experienced that trust was already present among the members where several of the GPs already knew each other (among other things the clusters were often based on existing professional fora), and that the professional discussions at the meetings were open and constructive. However, a few coordinators had observed that some members were reluctant to talk about their data and were inclined to take a defensive position if questioned about their clinical practice, particularly if the data showed large data variations among the clinics. Concerning discussions of data from the clinics, some coordinators reported that it could be challenging to facilitate the discussions at the meetings due to difficulties with assessing and interpreting the quantitative data presented at the meetings, e.g. with assessing the overall validity of the data, responding to objections about the quality of data voiced by other participants, and interpreting practice variations between clinics. These coordinators felt a need to be well-prepared for this aspect of the meetings and to increase their competencies in this area, so that they could better guide the discussions of data in the cluster:

*… to be able to [really] comprehend the meaning of these charts […] I mean, I can see that one clinic has made half as many services of some kind as another clinic, but what does that really mean?* (Cluster coordinator)

Many coordinators reported that the support from the regional quality units had been very useful in terms of presenting and interpreting data. The coordinators could either discuss data with the consultants from the regional quality units or have the regional consultants act as facilitators at the meeting (if the topic was within the competence area of the regional consultants).

### Attendance and engagement of cluster members

For the coordinators, attendance and engagement of the cluster members were important issues that affected many of their decisions about how to enact their role as coordinators. As described above, the coordinators tried to sustain and increase engagement in various ways, e.g. by a) shaping the activities of the cluster in line with the preferences of the members, b) initially selecting meeting-topics unlikely to cause controversy, c) using small group discussions. The coordinators generally found attendance rates to be high and that many cluster members engaged positively in the deliberations at the meetings, which made the role as coordinator easier:

*It [the commitment of the members] has been overwhelming. Of course, there are always some who are like: ‘these clusters, what’s that about?’ … but I actually think that most of them have been positive and have participated actively.* (Cluster coordinator)

The coordinators experienced that the autonomy of the clusters (in terms of choosing what issues to work with and how) was motivating for themselves and the other GPs. At the same time, some coordinators mentioned that the regulative pressure of the general agreement had helped to increase participation and attendance since it was assumed that most GPs preferred the clusters over some kind of accreditation.

However, in some clusters, a few members had been absent from the meetings, and in a few clusters the coordinators were not satisfied with attendance rates (about two thirds of the members attended the meetings in these clusters). While relatively rare, cases of continuing absenteeism were considered a challenge by the coordinators. Although the collective agreement stated it as a goal that all GPs should participate actively in a cluster, the formal framework did not provide a definition of ‘active participation’ or guidelines about how to deal with absenteeism. Hence, the clusters had to decide for themselves how to handle such issues. According to the coordinators there were different opinions among the GPs with some members requesting that monitoring of attendance and formal criteria for exclusion should be in place from the beginning (so that no one could free-ride by becoming a member of the cluster but not attend the meetings) while others preferred a more lenient approach where potential problems with absenteeism could be explored and discussed further if they occurred. As one coordinator described it:

*I register attendance […] but it’s difficult to say what the consequence [of absenteeism] should be […] and [there are] no definitions of participation. But the statutes [of the cluster] say that the general assembly can take up the matter of exclusion if someone does not attend [the meetings].* (Cluster coordinator)

The coordinators generally favoured a cautious and individual approach to absenteeism wanting first to explore the specific reasons why a member did not attend the meetings and if anything could be done to support attendance. Some coordinators expressed discomfort with the thought of having to reprimand some of their colleagues and thereby jeopardizing their relationship with them. Rather, the coordinators wanted to focus on making the cluster meetings attractive to the members.

## Discussion

In the formal framework for the quality clusters in Danish general practice, the cluster coordinators were given a central role in the development and operation of the clusters. From the perspective of the cluster coordinators, the most important enablers in the establishment process had been the positive attitudes and engagement of the GPs, the support from the regional quality units and KiAP, and the funding of the clusters which allowed for delegating administrative tasks and for remunerating the coordinators as well as external speakers and facilitators. At the same time, the development phase had been time-consuming and somewhat troublesome for the cluster coordinators due to the administrative tasks involved, and the many questions that had to be settled when translating the relatively open cluster concept into a specific, local cluster with broad appeal among the members. After having set up the clusters formally, the coordinators worked on making the professional meetings an attractive forum for learning and development. The coordinators were generally satisfied with the cluster meetings, but several coordinators also pointed to challenges in relation to the meetings, particularly with the obtainment of data and with the facilitation of peer discussions about the data. For some topics, it could be difficult to interpret the data, and some coordinators did not have previous training or experiences with facilitation.

Easy access to relevant and comprehensible data is crucial if the visions of data driven quality improvement in the clusters are to be realized. The clusters coordinators reported some challenges in this area, and experiences from Wales [4, 5] and Scotland [8] suggests that data obtainment and use is a difficult and ongoing issue. An evaluation from Wales reported that cluster leads felt that the use of patient-level data was still under-developed [4, 5] although the clusters were introduced in 2014. In a Scottish survey, only a fifth of the quality leads felt ‘fully’ or ‘almost fully’ supported with data while another fifth felt ‘not at all supported’ [8]. Since the time of our study, the Danish web-portal for extracting medication data has been updated (Ordiprax +). This will provide new opportunities for the clusters. However, for other topics, data challenges remain. For example, obtaining data related to the interfaces between general practice and the municipalities was experienced particularly difficult by the cluster coordinators. Formalizing communication via a regular contact person in the municipality (or utilizing existing collaborative structures) could be a way to create easier access to data and perhaps to developing municipality related data packages for the clusters.

Research on quality circles has shown that facilitation is crucial for the success of collective improvement work [6]. Rohrbasser et al. ([6], p. 3) describes how the task of the facilitator is to “open discussions, summarise, clarify statements and question issues”, and to create an atmosphere of trust where participants can openly share their practice and compare it with various kinds of evidence. This can be a difficult role, which requires “abilities and skills to manage group dynamics” ([6], p. 3). However, prior to the establishment of the clusters, the cluster coordinators had not received specific training in facilitation of such group discussions and the coordinators had very different prerequisites for enacting this role. Furthermore, considering the challenge of analysing and acting on data, previous studies have pointed out that health professionals often lack the necessary time and skills to engage effectively in data driven improvement efforts based on audit and feedback [13]. A recent report from the UK showed that only 20% of the GPs surveyed were familiar with the PDSA-Cycle (Plan-Do-Study-Act) and that 42% of the GPs identified “a lack of skills in managing and analysing data as being a barrier to improvement in general practice” ([14], p. 6). This underlines the importance of providing training opportunities in facilitation, quality improvement techniques, and data analysis for the cluster coordinators (or other cluster members) and/or of making external facilitation offers widely available for the clusters. Future studies could explore issues of facilitation and improvement techniques in the clusters in terms of variations, challenges, and needs.

After the official decision to introduce clusters in general practice had been announced, the GPs relatively rapidly established clusters across the country with high rates of membership. Further, the cluster coordinators in our study were generally satisfied with the cluster meetings and the engagement of the members in the cluster activities. A high level of member participation and engagement is not only a precondition if the clusters activities are to have a wide impact upon the internal processes of quality improvement in the clinics but also if the activities are to improve cross-sectorial collaborations involving general practice. Previous research has suggested that it can be difficult to mobilize widespread support for cross-sectorial collaboration among the GPs when the task of mobilization is delegated to individual practitioners without an underlying framework of support [15], but the cluster framework may offer new opportunities. Hence, if the clusters are able to mobilize large numbers of GPs on a continuing basis, they may become important units for collaboration between general practice and other parts of the health care system. However, a high level of engagement over time cannot be taken for granted. A report on general practice clusters from Wales pointed to problems with participation and engagement among the GPs due to time pressure in the clinic [16]. Hence, it will be interesting to see if a high level of engagement among the Danish GPs can be sustained over time. Engagement will presumably be affected by the extent to which the GPs perceive a positive impact of the clusters compared to the time investments they make. In a survey from Scotland, Mercer et al. [2020] concluded with concern that the GPs generally perceived “little or no improvements in various aspects of the quality of care they deliver as a result of clusters” [8]. We are pursuing questions of impact and engagement in the Danish clusters in ongoing studies.

Finally, it was interesting that a few clusters in our study had chosen not to use the cluster packages offered by the regional and national agencies since they believed that doing so would subject the clusters to unwarranted public interference. This suggests that a certain tension exists in the field where some GPs are concerned about the regulatory implications of the clusters even though the official policy is that the clusters shall have a high level of freedom and that cluster data shall only be used for local improvement (and not for external control and sanctioning). In the context of patient safety in the National Health Service (NHS), Armstrong et al. [17] found that it can be difficult to convince professionals that new programs for quality and safety aim for improvement and not for accountability in a health care field where a logic of accountability has been influential for many years. The extent and nature of such a tension in the context of general practice clusters could be explored in future studies.

### Limitations

Although we believe to have reached a reasonable level of data saturation and the sample was relatively large (with the coordinators representing approximately a quarter of the clusters that existed at the beginning of the study), the study may have some limitations. Thus, we explored the challenges and enablers in the process of developing and operating the clusters from the perspective of the cluster coordinators, and the study was conducted relatively shortly after the establishment of the clusters. Hence, other barriers to – or enablers for – data driven quality improvement in the clusters than those experienced by the cluster coordinators may exist, and some of these may be more obvious when the clusters have been operating for a longer period of time. Investigating the perspectives and experiences of ordinary members and external stakeholders will shed more light on the challenges and opportunities for realizing the visions for the clusters.

## Conclusion

In spite of some administrative challenges, the coordinators were generally satisfied with the process of developing the general practice clusters – a process which had progressed relatively fast with widespread participation from the GPs and support from the formal framework set-up in relation to the clusters. The findings from the study also suggest that operative challenges related to data obtainment, data analysis, and facilitation will have to be addressed ongoingly. Future research should look closer into how the clusters work with quality improvement in terms of approaches and methods, how the members perceive and respond to these approaches, and how the cluster work affects clinical practice and collaborative relations between general practice and other health care providers.

## Supplementary Information

**Additional file 1.** Consolidated criteria for reporting qualitative studies (COREQ): 32-item checklist.

## Data Availability

Anonymised interview transcripts can be made available from the corresponding author upon reasonable request.

## Abbreviations

GP: General practitioner
KiAP: Quality in general practice
PLO: Organisation of general practitioners
