# Supplementary material for: Introducing quality clusters in general practice – a qualitative study of the experiences of cluster coordinators
Source: BMC Prim Care. 2022 Aug 25;23:215. doi: 10.1186/s12875-022-01828-2 (PMC9404612; doi:10.1186/s12875-022-01828-2)
Supplement: Supplementary file 1 — Additional file 1. Consolidated criteria for reporting qualitative studies (COREQ): 32-item checklist. [file 12875_2022_1828_MOESM1_ESM.docx]

**Consolidated criteria for reporting qualitative studies (COREQ): 32-item checklist**

| **Topic/Domain + Item no. + questions** | **Authors’ comments** | **Reported on page no** |
| --- | --- | --- |
| **Domain 1: Research team and reflexivity** |  |  |
| **Personal Characteristics** |  |  |
| 1. Interviewer/facilitator: Which author/s conducted the interview or focus group? | The interviews were conducted by MBK, MB, THM, MTK, MHM and MBOK. In the section on Authors’ contributions it appears who collected the data. | 27 |
| 2. Credentials: What were the researcher’s credentials? E.g. PhD, MD | Five members of the research team had a background in social science (MBK, THM, MBOK, MHM, PK) and three members were medical doctors (MB, MTK, JS), two of whom were GPs (MTK, JS). | 11 |
| 3. Occupation: What was their occupation at the time of the study? | The researchers were employed at The Research Unit for General Practice in Copenhagen, The Research Unit for General Practice in Odense or at VIVE – The Danish Center for Social Science Research. | 1 (title page) |
| 4. Gender: Was the researcher male or female? | MHM, PK, MB: Female  MBK, THM, MBOK, MTK, JS: Male | 1 (names on title page) |
| 5. Experience and training: What experience or training did the researcher have? | Five members of the research team had a background in social science (MBK, THM, MBOK, MHM, PK) while three members were medical doctors (MB, MTK, JS), two of whom were GPs (MTK, JS). | 11 |
| **Relationship with participants** |  |  |
| 6. Relationship established: Was a relationship established prior to study commencement? | No | NA |
| 7. Participant knowledge of the interviewer: What did the participants know about the researcher? e.g. personal goals, reasons for doing the research | It is stated that all informants gave their informed consent to participate (hence the participants were informed about the subject of the study and the interviews and about the institutional affiliation of the study) | 26 |
| 8. Interviewer characteristics: What characteristics were reported about the interviewer/facilitator? e.g. Bias, assumptions, reasons and interests in the research topic | See point no. 5.  The reasons for doing the study are explained in the introduction of the paper. | 4-5, 11 |
| **Domain 2: study design** |  |  |
| **Theoretical framework** |  |  |
| 9. Methodological orientation and theory: What methodological orientation was stated to underpin the study? e.g. grounded theory, discourse analysis, ethnography, phenomenology, content analysis | Research on quality improvement and quality circles in general practice inspired the research questions and the analysis. A qualitative thematic approach was used to analyze the interviews. | 10 |
| **Participant selection** |  |  |
| 10. Sampling: How were participants selected? e.g. purposive, convenience, consecutive, snowball | Purposive sampling | 8-9 |
| 11. Method of approach: How were participants approached? e.g. face-to-face, telephone, mail, email | Invitations and appointments were handled via e-mail (in some cases supplemented by a telephone call) | 9 |
| 12. Sample size: How many participants were in the study? | 25 | 8 |
| 13. Non-participation: How many people refused to participate or dropped out? Reasons? | Three of the invited coordinators declined to participate. Two of these replied that they did not have time to participate, and one coordinator declined without providing a reason. | 9 |
| **Setting** |  |  |
| 14. Setting of data collection: Where was the data collected? e.g. home, clinic, workplace | Most interviews were performed in the clinic of the coordinator, but some were performed at the venues of the cluster meetings. | 9 |
| 15. Presence of non-participants: Was anyone else present besides the participants and researchers? | No | NA |
| 16. Description of sample: What are the important characteristics of the sample? e.g. demographic data | All participants were active as GPs who had taken on the role of cluster coordinator. The participants came from clusters that had carried out at least two meetings on a specific professional topic. They also represented clusters of varying sizes and different geographic locations. | 8-9 |
| **Data collection** |  |  |
| 17. Interview guide: Were questions, prompts, guides provided by the authors? Was it pilot tested? | The topics contained in the interview guide are presented in the methods section. One pilot interview was performed. | 9-10 |
| 18. Repeat interviews: Were repeat interviews carried out? If yes, how many? | No | NA |
| 19. Audio/visual recording: Did the research use audio or visual recording to collect the data? | All interviews were audio recorded. | 9 |
| 20. Field notes: Were field notes made during and/or after the interview or focus group? | Some notes were made but they were not used in the analysis for this paper. | NA |
| 21. Duration: What was the duration of the interviews or focus group? | 45-90 minutes | 9 |
| 22. Data saturation: Was data saturation discussed? | Yes | 22 |
| 23. Transcripts returned: Were transcripts returned to participants for comment and/or correction? | No | NA |
| **Domain 3: analysis and findings** |  |  |
| **Data analysis** |  |  |
| 24. Number of data coders: How many data coders coded the data? | Five |  |
| 25. Description of the coding tree: Did authors provide a description of the coding tree? | No, but the major topics (from the interview guide) that were used to code the data are described. | (10) |
| 26. Derivation of themes: Were themes identified in advance or derived from the data? | We coded the transcribed interviews with reference to the central themes of the interview guide while also being open to emerging themes. | 10 |
| 27. Software: What software, if applicable, was used to manage the data? | NVivo and Microsoft Word | 10 |
| 28. Participant checking: Did participants provide feedback on the findings? | No | NA |
| **Reporting** |  |  |
| 29. Quotations presented: Were participant quotations presented to illustrate the themes/findings? Was each quotation identified? e.g. participant number | Yes (but not numbered since quotations come from different participants) | 10 |
| 30. Data and findings consistent: Was there consistency between the data presented and the findings? | Yes | 11-19 |
| 31. Clarity of major themes: Were major themes clearly presented in the findings? | Yes | 11-19 |
| 32. Clarity of minor themes: Is there a description of diverse cases or discussion of minor themes? | The paper mainly focuses on the major themes related to the aim of the study, articulating how the coordinators generally enacted and experienced their role. However, experiences diverting from the general picture are also presented (using formulations such “a few coordinators reported that…” or “a few coordinators had observed that…”). | 15-18 |
